# Oral Health-Related Quality of Life Appraised by OHIP-14 Between Urban and Rural Areas in Kutai Kartanegara Regency, Indonesia: Pilot Pathfinder Survey

**DOI:** 10.2174/1874210601711010557

**Published:** 2017-10-31

**Authors:** Fuad Akbar Husain, Fransiske Tatengkeng

**Affiliations:** 1 Department of Dental Public Health, Faculty of Dentistry, Hasanuddin University, Makassar, Indonesia; 2 Faculty of Dentistry, Hasanuddin University, Makassar, Indonesia

**Keywords:** Oral health-related quality of life, OHIP-14, OHRQoL in urban and rural, QoL in Indonesia, QoL in urban areas, QoL in rural areas

## Abstract

**Background:**

Health-Related Quality of Life (HRQoL) periphrastically has a significant impact on oral health. A recent study has shown the significant facts of the oral health-related quality of life based on many factors such as individual, social status, household management, daily habits, and local factors. The differences in the oral health status possibly occur in between countries, different regions, and topographical areas frequently and indirectly contributing to oral health status.

**Objective:**

The objective is to evaluate the difference of Oral Health-Related Quality of Life (OHRQoL) and to assess the main affected dimension between rural and urban areas in Kutai Kartanegara Regency.

**Methods:**

This study uses pilot pathfinder design. The respondents comprised of 214 adults who were elder than 18 years and were randomly selected from urban and rural areas in Kutai Kartanegara Regency, Indonesia. The data were collected by 103 samples from the rural area and 111 respondents from the urban area. Oral Health Impacts Profile (OHIP-14) has been translated to Bahasa (Indonesia version). OHIP-14 was used to assess the subjects’ oral health-related impact. Shapiro-Wilk and Mann Whitney tests were used to analyze the data, and *p*-value was set at *P* < 0.05.

**Results:**

The mean OHIP scores in the urban and the rural areas were 25.4 and 28.8, respectively. The overall OHIP-14 score showed a significant statistical difference *P*= 0,009 (*P* < 0.05) between rural and urban area.

**Conclusion:**

This study illustrates that oral health-related quality of life in the urban area is better than in the rural area. Physical pain components of the OHRQoL are the major oral problems associated with both the areas.

## 1 INTRODUCTION

Oral health is the main component of individuals’ general health and it allows individuals to run their daily activities (mastication, articulation, socializing) without any illness, discomfort, and disability. Moreover, a systemic disease is somehow related to many oral manifestations and reflected by the individual quality of life (QoL) [1-3]. Oral health also has a main contribution to the quality of life, and it directly affects people at their physical, cognitive, emotional, and social level [4]. Based on Locker’s study on Zuccolo, it has been shown that oral health affects people physically and psychologically due to many aspects such as their way of life, interaction with each other, their social well-being *etc*. [3].

The key to improve QoL is by repatriating oral function, mastication, preventing oral disease, repairing oral tissue, and tackling the patient’s complaints [2-5]. To measure the Oral Health-Related Quality of Life (OHRQoL), Slade and Spencer in 1994 tested the performance to measure the functional, social, and psychological outcomes of oral conditions based on 49 questions known as OHIP-49. The OHRQoL’s measure (OHIP) is a suitable subjective indicator that provides information about the impacts of oral conditions on an individual’s life and perceived need for dental treatment. The Oral Health Impact Profile (OHIP) is a questionnaire that measures people’s perception of the social impact of oral disorders on their well-being. Slade in 1997 developed a short-form of it with 14 questions, named OHIP-14 which showed good reliability, validity and precision. Fourteen items of OHIP are divided into seven dimensions; functional limitation, physical discomfort, psychological discomfort, physical disability, psychological disability, social disability and handicaps. This OHIP-14 has been widely used across the world for various research purposes with modifications including language and regional concerns. Assessing the adult oral health-related quality of life is one of the primary needs [1, 4, 1-8].

One of the most crucial periods to prevent dental disease begins in adolescence which also affects the future of oral health. The World Health Organization in 2012 in Galloway suggests that the correlation between oral health-related quality of life (OHRQoL) and well-being was influenced by six factors; physical, environment, social relationships, physiological, independence stage and spiritual life. The result of the empirical analysis suggested that the impact of the quality of life relates to social demographic data (age and gender) along with economic and epidemiological aspects of culture [1, 9, 10].

An epidemiological survey in Greece in 2009 in Papaioannou showed the significant differences in quality of life found in each region as well as the differences in oral conditions between communities living in urban and rural areas [4]. A research by Spelberg in Goran (2006) in Germany discovered that the quality of life of people in rural areas was better as compared to urban setting due to fresh water, fresh air, and eco-friendly spaces in rural areas contrary to unhealthy atmosphere resulting from city’s infrastructure. However, there are several other factors that influence other than geographical location. By noticing those differences, the author also compared the impact of individual oral status on the OHRQoL by different regions [4,11-13].

According to the statistical data of Kutai Regency obtained in 2006, Tenggarong subdistrict which is situated at the center of Kutai Kartanegara Regency has 114,307 inhabitants. This number is far above the inhabitants of Samboja subdistrict located in the coast which only has a population of 63,467 people. Therefore, by considering the population density, Tenggarong subdistrict is considered an urban area while Samboja subdistrict is considered as a rural area. On the other hand, 700 people (0.6% of the total population) living in Tenggarong subdistrict were observed to be farmers, 708 (0.6%) as fishermen, while the majority were found to be civil servants being 7965 people registered with the peak of the percentage 6.9%. In contrast, in Samboja subdistrict , majority were farmers, comprising 4358 people (6.8% of the total population), while 2926 (4.6%) people were fishermen where the civil servants were only 952 (1.4%). [14-16]

### 1.1 Statement of Problem

Remote area and geographical differences could not be denied showing the significant difference scores and quality of life in both communities as well as in each region. In terms of livelihoods, Tenggarong subdistrict was observed to be dominated by civil servants while Samboja subdistrict has the majority of farmers. This trend affected the economic growth of each region and their role in improving the quality of life. Based on the aforementioned situation, the researcher was interested in evaluating the differences in the oral health-related quality of life (OHRQoL) in different regions and in assessing the main affected dimension of rural and urban areas in Kutai Kartanegara Regency.

## 2 MATERIALS AND METHODS

This study used pilot pathfinder design. The study respondents comprised of 214 adults with age more than 18, and were randomly selected from urban and rural areas of Kutai Kartanegara Regency. The urban area refers to Tenggarong subdistrict while the rural area refers to Samboja subdistrict . The inclusion criteria was adults with of more than 18 years willing to fill the questionnaire, while the respondents who were unable to fill in all the general data as well as to return the questionnaire were excluded. The survey was conducted at three different health centers located in Tenggarong district (urban) and another three including one additional hospital in Samboja district (rural). Among 214 respondents in total, eventually there were only 202 valid respondents left with 96 respondents from urban areas and 106 from rural areas.

Determination of the respondents is based on the report by WHO which is according to the geographical area covering large populations and very complex health systems. The relevant and reliable information can be obtained by estimating the number of appropriate respondents based on the age category in urban and rural. The final result shows that both urban and rural areas at least had 100 respondents. The assessment of Quality of Life was carried out using OHIP-14 questionnaire that was simultaneously translated into Indonesia version. OHIP-14 questionnaire includes seven dimensions with 14 items to determine the quality of life. The higher the average value of the seven dimensions, the more negative the impact of oral health on the quality of life of an individual. The seven dimensions include functional limitations, physical pain, psychological discomfort, physical disability, psychological disability, social disability and handicap. On the dimensions that influence the quality of life, the combination of alternative answers like “often” and “very often” was a reference to determine whether those dimensions have a negative impact on people's quality of life. Classification of a quality of life was made as good, moderate and severe. The survey data were organised and analysed by using *SPSS* ver.18 for Windows platform (*SPSS* Inc., Chicago, Illinois, USA) and MS. Excel (Microsoft Office, Windows 2007, USA). Differences were considered significant when *p <* 0.05.

## 3 RESULTS

A total of 202 respondents (96 from urban and 106 from rural) participated in the survey. The distribution of respondents according to demographic / characteristic respondent among rural and urban areas is presented in Table **1**.

Table **1** illustrates that both the areas were dominated by women being 77.1% in the rural areas and 75.5% in the urban areas. In terms of age, the range of 18-44 years was the highest for both urban and rural areas with percentage of 38% and 61.5%. Two age-groups between 18-44 years and also over 44 years were considered based on WHO’s category of adult age. In particular categorisation, age between 18-44 years was categorised as the age of a young adult while 44 years was categorised as the age of an adult.

The education level of the respondents in urban and rural areas was high school graduates being 57.3% in urban areas and 42.1% in rural areas. However, the respondents living in rural areas who did not have any occupation accounted for 33% while in urban areas, the value was 27%.

Monthly income on revenue of USD 0-7.5 showed the highest number of respondents in rural areas with a percentage of 79.2% while being 52.1% with a monthly income for more than USD 75.

Table **2** shows that 41 respondents (20.3%) responded to the question of “ever felt difficulty in pronouncing any words because of the problems with your teeth or mouth” as “often”, and very few people (7 (3.5%))responded to the question “totally unable to function because of the problem with your teeth or mouth”. On the question “ever feel worried / anxious because of the problem in the oral cavity” the alternative answer “very often” was the least, with only a few numbers of respondents. The question of “felt totally unable to function because of the problem with your teeth or mouth” had the lowest mean value with a mean of 1.65, which means that almost all respondents answered rarely and the highest mean value correlated to the question “uncomfortable to eat any food because of the problem with your teeth or mouth” with a mean value of 2.25 which means the respondents answered in average sometimes feel uncomfortable while chewing due to the problem in the oral cavity.

Table **3** shows that the mean value in urban areas was the highest on the dimension of physical pain with mean value of 4.09, while the lowest value was in the dimension of disability with a mean value of 3.19. Moreover, the mean value was the highest in rural area with the dimension of psychological discomfort with a mean value of 4.47 and the lowest on the dimension of delay with a mean of 3.57.

Table **3** also shows a significant difference between the two regions with *p* = 0.009 (*p* <0.005) with a higher total of OHIP-14 score in urban areas. This shows that the quality of life in rural areas is worse.

In addition, Table **3** illustrates the significant difference between urban and rural areas (*p* <0.05) in the functional dimension limitations with *p* = 0.027, dimension of ill physical condition with *p* value = 0.037 and dimension of psychic disability with *p* value = 0.018. Among these three dimensions, the average mean value in rural areas was throughout higher than urban areas. Therefore, rural areas were observed to have a lower quality of life compared to urban areas.

Table **4** shows the differences in the value of the dimensions of the quality of life between urban and rural in which the impact of low quality of life is classified when one item of question is answered with “often” and “very often” alternative answers. On the question about the “trouble pronouncing” there were as many as 27.2% respondents whose quality of life was affected. Moreover, the lowest percentage (5.4%) of respondents felt difficulty due to poor oral cavity affecting their quality of life.

Impact Profile questionnaire. *Impacts reported “fairly often” or “very often” in preceding three months.

## 4 DISCUSSION

The result presented in this study, show a significant difference between the rural and urban areas in terms of affected oral health as well as the differences in the quality of life. The higher score of OHIP in rural areas indicates that the quality of life in rural areas in the worst compared to urban areas. The same results shown in a recent study by Carneiro in 2010 on rural areas of Brazil suggest that the more remote the area, the more it affects the quality of life. It suggests that the high score of OHIP will always be equal to the negative impact of the quality of life. This is in line with a research conducted by Papaioannou in 2015 in urban and rural areas showing that the rural areas had a higher mean value of OHIP-14 than urban areas, both of which are consistent with the survey results showing a higher mean value of OHIP-14 in rural areas. Dams indicate that rural areas have poor oral health and over all quality of life of the individual; this is attributed to the lack of facilities in the area, very fewer medical personnel than the number of residents that poses problems in obtaining proper services to help maintain the quality of life [1, 17].

In terms of respondent’s education, it was discovered that the education level of a person influences his/her quality of life. A research conducted by Papaioannou showed the difference in OHIP value based on public education level. The research showed high school graduates with lower OHIP value compared to scholars. Those living in urban areas had lower OHIP scores than those in rural areas. [1].

Similarly, a study conducted by Marilia in 2014, analysing the highest OHIP score resulting in low quality of life of the respondents who received education in more than nine years depicted the number of such respondents to be lower as compared to those who had good quality of life with lower OHIP score which comprised of 196 respondents with 9-11 years of educational experience, equal to junior or senior high school graduates. Moreover, those respondents who studied over than 11 years being university or college graduates, showed the lowest number of OHIP score; this trend, leads to the very good quality of life [18].

The level of education related to oral health is very important, which is also justified by a research conducted by Ahmed, showing better oral health of people who have received education on oral health than those who did not. This implies that higher level of a person's education helps in increasing the awareness about oral health [1, 2, 19, 20]. Respondents who were not qualified enough i.e. graduated from elementary and junior high school were 23 in urban areas and 54 in number in rural areas. Therefore, the quality of life in Tenggarong District was observed to be better than in Samboja District. This suggests that the education level also affects the quality of life.

Monthly income is an important factor in determining the quality of life. A study in Hudacova in 2010 showed that there exists a relationship between OHIP and a person's income. This was confirmed by a study in Biazevic showing that 71.2% of those with high incomes showed low OHIP scores, suggesting better quality of life than being 39.9% of those with low incomes and poor oral health [21, 22]. The results of this study showed that in rural areas, people have low income with livelihoods as farmers resulting in higher OHIP rates than those in urban areas with a profession dominated by office workers, being self-employed and private employees [14, 21, 22] The quality of life is also influenced by the location or area of origin of the community, therefore, rural and urban areas have differences in the quality of life. In this study, the quality of life associated with oral health showed a worst impact on rural areas represented by Samboja subdistrict compared to those living in urban areas represented by Tenggarong subdistrict due to several aspects such as education, availability of health services, income, livelihoods and age. Other aspects such as dental care, periodontal disease and tooth loss resulting in functional limitations in the oral cavity also contribute to this. However, the quality of one's life can possibly change to better if all the limitations are addressed and mastication function is restored as before [22-26].

## CONCLUSION

For the representative respondents of Kutai Kartanegara Regency who took part in the present study, the quality of life associated with oral health showed good quality of life in urban areas represented by Tenggarong subdistrict being significantly different from the rural areas represented by Samboja subdistrict . Further investigation of the Oral Health Quality of Life of individuals with a significant sign of disease, current or past, must be undertaken. Another aspect that needs to be evaluated is the correlation between Oral Health Quality of Life and ages, sex, education level, income, and livelihood. The data from such studies aid in advocating the formation of a suitable profile for future dentists to handle an ageing population, and also to provide additional data for dental clinics as well as underline the need in providing the necessary resources and public funds for dentistry. It is important to place dental and oral health in the proper context.

## Figures and Tables

**Table 1:** Distribution of respondents according to demographic/characteristic among rural and urban areas.

Respondents Characteristic	Rural		Urban	
	Frequency (n)	Percent (%)	Frequency (n)	Percent (%)
**Sex**				
Male	22	22.9	26	24,5
Female	74	77.1	80	75,5
**Age**				
18-44 Years	59	61.5	68	38
>44 Years	37	38.5	64.2	35.8
**Educational Level**				
No Education	6	6.3	19	12.4
Elementary School	9	9.4	19	13.9
Junior High School	8	8.3	16	11.9
Senior High School	55	57.3	30	42.1
Graduated	18	18.8	22	19.8
**Occupation**				
Jobless	27	28.1	35	33
Farmer	6	6.3	8	7.5
Plumbers	0	0	4	3.8
Students	2	2.1	0	0
Entrepreneur	10	10.4	7	6.6
Officer	10	10.4	7	6.6
Government Employees	14	14.6	23	21.7
Etc.	27	28.1	22	20.8
**Monthly Income**				
USD 0-7.5 (Lower Class)	39	40.6	84	79.2
USD 7.6- 37.5 (Lower Middle)	4	3.8	4	3.8
USD 37.6-75 (Middle Class)	3	3.1	0	0
USD > 75 (Upper Class)	50	52.1	18	17
Total	96	100	106	100

**Tabel 2:** Distribution of responses for OHIP score among rural and urban citizen.

List of Question on OHIP-14 Questioner	Responses					Mean (sd)
Never	Seldom	Sometimes	Often	Always
n (%)	n (%)	n (%)	n (%)	n (%)
Have you had trouble pronouncing any words because of problems with your teeth or mouth	0 (0%)	111 (55%)	36 17.8%)	41 (20.3%)	14 (6.9%)	1.79 (0.99)
Have you felt that your sense of taste has worsened because of problems with your teeth or mouth	106 (52.5%)	46 (22.8%)	38 (18.8)	9 (4.5)	3 (1.5)	1.80 (0.99)
Have you had painful aching in your mouth	73 (36.1%)	55 (27.2%)	60 (29.7%)	12 (5.9%)	2 (1.0%)	2.08 (0.99)
Have you found it uncomfortable to eat any foods because of problems with your teeth or mouth	65 (32.2%)	50 (24.8%)	64 (31.7%)	18 (8.9%)	5 (2.5%)	2.25 (1.07)
Have you been self-conscious because of your teeth or mouth	78 (38.6%)	32 (15.8%)	66 (32.7%)	25 (12.4%)	1 (0.5%)	2.20 (1.10)
Have you felt tense because of problems with your teeth or mouth	95 (47%)	44 (21.8%)	43 (21.3%)	18 (8.9%)	2 (1.0%)	1.95 (1.06)
Has been your diet been unsatisfactory because of problems with your teeth of mouth	87 (43.1%)	41 (20.3%)	48 (23.8%)	18 (8.9%)	2 (1.0%)	2.09 (1.14)
Have you had to interrupt meals because of problems with your teeth or mouth?	79 (39.1%)	53 (26.2%)	46 (22.8%)	20 (9.9%)	4 (2.0%)	2.09 (1.09)
Have you found it difficult to relax because of problems with your teeth or mouth	94 (46.5%)	51 (25.2%)	39 (19.3)	16 (7.9)	2 (1.0%)	1.92 (1.03)
Have you been a bit embarrassed because of problems with your teeth or mouth	88 (43.6%)	42 (20.8%)	42 (20.8%)	24 911.9%)	6 (3.0%)	2.10 (1.17)
Have you been a bit irritable with other people because of problems with your teeth or mouth	120 (59.4%)	39 (19.3%)	26 (12.9%)	15 (7.4%)	2 (1.0%)	1.71 (1.01)
Have you had difficulty doing your usual jobs because of problems with your teeth or mouth	115 (56.99%)	45 (22.3%)	30 (14.9%)	9 (4.5%)	3 (1.5%)	1.71 (0.97)
Have you felt that life in general was less satisfying because of problems with your teeth or mouth	119 (58.9%)	43 (21.3%)	24 (11.9%)	11 (5.4%)	5 (2.5%)	1.71 (1.03)
Have you been totally unable to function because of problems with your teeth or mouth	122 (60.4%)	43 (21.3)	26 (12.9%)	7 (3.5%)	4 (2.0%)	1.65 (0.96)

**Table 3:** Mean OHIP-14 scores according to urban and rural area.

Mean (SD)			
	Urban	Rural	*P*
Functional Limitation	3.30 (1.66)	3.85 (1.81)	0.027*
Physical Pain	4.09 (1.9)	4.58 (1.86)	0.037*
Psychological Discomfort	3.88 (1.87)	4.47 (2.16)	0.052
Psychological Disability	3.96 (2.00)	4.42 (2.07)	0.094
Physical Disability	3.71 (1.80)	4.38 (2.04)	0.018*
Social Handicap	3.27 (1.76)	3.60 (1.88)	0.203
Handicap	3.19 (1.82)	3.57 (1.96)	0.150
Total OHIP Score	25.4(10.84)	28.8(10.67)	0.009*

*Mann-withney test: p<0.05; significant

**Table 4:** Prevalence of adverse impacts on quality of life prior to area*.

Impact Experienced due to Problems with Teeth, Mouth or Dentures	n of Patients Reporting Impact*	% of Patients Reporting impact*
Trouble Pronouncing	55	27.2
Taste Affected	12	5.9
Painful Aching	14	6.9
Uncomfortable to Eat	23	11.4
Been Self Conscious	26	12.9
Felt Tense	20	9.9
Diet Unsatisfactory	20	9.9
Interrupted Meals	24	11.9
Difficult to Relax	18	8.9
Been Embarrassed	30	14.9
Been a Bit Irritable	17	8.4
Difficult Doing Jobs	12	5.9
Life Less Satisfying	16	7.9
Unable to Function	11	5.4

n = 202 subjects in urban and rural area

## LIST OF ABBREVIATIONS

WHO: World Health Organization
QoL: Quality of Life
OHRQoL: Oral Health-Related Quality of Life
HRQoL: Health-Related Quality of Life
OHIP: Oral Health Impact Profile
